# Cumulative asbestos exposure and mortality from asbestos related diseases in a pooled analysis of 21 asbestos cement cohorts in Italy

**DOI:** 10.1186/s12940-019-0510-6

**Published:** 2019-08-07

**Authors:** Ferdinando Luberto, Daniela Ferrante, Stefano Silvestri, Alessia Angelini, Francesco Cuccaro, Anna Maria Nannavecchia, Enrico Oddone, Massimo Vicentini, Francesco Barone-Adesi, Tiziana Cena, Dario Mirabelli, Lucia Mangone, Francesca Roncaglia, Orietta Sala, Simona Menegozzo, Roberta Pirastu, Danila Azzolina, Sara Tunesi, Elisabetta Chellini, Lucia Miligi, Patrizia Perticaroli, Aldo Pettinari, Vittoria Bressan, Enzo Merler, Paolo Girardi, Lucia Bisceglia, Alessandro Marinaccio, Stefania Massari, Corrado Magnani, the working group, Lisa Bauleo, Antonio Baldassarre, Carol Brentisci, Barbara Cortini, Stefania Curti, Manuela Gangemi, Giuseppe Gorini, Patrizia Legittimo, Francesco Marinelli, Pasqualina Marinilli, Stefano Mattioli, Marina Musti, Chiara Panato, Venere Leda Mara Pavone, Alessandra Ranucci, Elisa Romeo, Corrado Scarnato, Cinzia Storchi, Antonella Stura, Simona Verdi

**Affiliations:** 1 Epidemiology Unit, Azienda Unità Sanitaria Locale - IRCCS di Reggio Emilia, Reggio Emilia, Italy; 2 Unit of Medical Statistics and Cancer Epidemiology, Department of Translational Medicine, University of Eastern Piedmont, via Solaroli 17, 28100 Novara, Italy; 3 CPO-Piedmont, Novara, Italy; 4 Unit of Epidemiology and Statistics, Local Health Unit of Barletta-Andria-Trani, Barletta, Italy; 5 Department of Public Health, Experimental and Forensic Medicine, University of Pavia, and ICS Maugeri IRCCS, Pavia, Italy; 6 Department of Pharmaceutical Sciences, University of Eastern Piedmont, and CPO Piedmont, Novara, Italy; 7 Unit of Cancer Epidemiology, CPO Piedmont and University of Turin, Turin, Italy; 8 Regional Agency for Prevention, Environment and Energy Emilia-Romagna, Provincial Office of Reggio Emilia, Reggio Emilia, Italy; 9 National Cancer Institute IRCCS Fondazione Pascale, Naples, Italy; 10 Department of Biology and Biotechnologies “Charles Darwin”, Sapienza University, Rome, Italy; 11 Occupational & Environmental Epidemiology Unit - Institute for Cancer Research, Prevention and Clinical Network (ISPRO), Florence, Italy; 12 Prevention Department, ASUR Marche, Senigallia, Italy; 13 UOSD Servizio di Epidemiologia AULSS6 EUGANEA, Padua, Italy; 14 Mesothelioma Register of the Veneto Region, Regional Epidemiologic System, Local Health Unit 6, Padua, Italy; 15 Apulia Regional Agency for Health and Social Policies - ARESS Puglia, Bari, Italy; 16 Italian Workers’ Compensation Authority (INAIL), Department of Occupational and Environmental Medicine, Epidemiology and Hygiene, Unit of Occupational and Environmental Epidemiology, Italian Mesothelioma Register, Rome, Italy; 17 Interdepartmental Centre G. Scansetti for Studies on Asbestos and other Toxic Particulates, University of Turin, Turin, Italy

**Keywords:** Asbestos, Asbestos-cement, Dose response relationship, Mesothelioma, Lung cancer, Ovarian Cancer, Epidemiology

## Abstract

**Background:**

Despite the available information on cancer risk, asbestos is used in large areas in the world, mostly in the production of asbestos cement. Moreover, questions are raised regarding the shape of the dose response relation, the relation with time since exposure and the association with neoplasms in various organs. We conducted a study on the relationship between cumulative asbestos exposure and mortality from asbestos related diseases in a large Italian pool of 21 cohorts of asbestos-cement workers with protracted exposure to both chrysotile and amphibole asbestos.

**Methods:**

The cohort included 13,076 workers, 81.9% men and 18.1% women, working in 21 Italian asbestos-cement factories, with over 40 years of observation. Exposure was estimated by plant and period, and weighted for the type of asbestos used. Data were analysed with consideration of cause of death, cumulative exposure and time since first exposure (TSFE), and by gender. SMRs were computed using reference rates by region, gender and calendar time. Poisson regression models including cubic splines were used to analyse the effect of cumulative exposure to asbestos and TSFE on mortality for asbestos-related diseases. 95% Confidence Intervals (CI) were computed according to the Poisson distribution.

**Results:**

Mortality was significantly increased for ‘All Causes’ and ‘All Malignant Neoplasm (MN)’, in both genders. Considering asbestos related diseases (ARDs), statistically significant excesses were observed for MN of peritoneum (SMR: men 14.19; women 15.14), pleura (SMR: 22.35 and 48.10), lung (SMR: 1.67 and 1.67), ovary (in the highest exposure class SMR 2.45), and asbestosis (SMR: 507 and 1023). Mortality for ARDs, in particular pleural and peritoneal malignancies, lung cancer, ovarian cancer and asbestosis increased monotonically with cumulative exposure. Pleural MN mortality increased progressively in the first 40 years of TSFE, then reached a plateau, while peritoneal MN showed a continuous increase. The trend of lung cancer SMRs also showed a flattening after 40 years of TSFE. Attributable proportions for pleural, peritoneal, and lung MN were respectively 96, 93 and 40%.

**Conclusions:**

Mortality for ARDs was associated with cumulative exposure to asbestos. Risk of death from pleural MN did not increase indefinitely with TSFE but eventually reached a plateau, consistently with reports from other recent studies.

**Electronic supplementary material:**

The online version of this article (10.1186/s12940-019-0510-6) contains supplementary material, which is available to authorized users.

## Introduction

Asbestos fibres in their different mineralogical forms of chrysotile and amphiboles are a well known carcinogen acting on the respiratory tract and other organs. In 2009, the International Agency for Research on Cancer (IARC) updated the evaluation of asbestos fibres and confirmed that all types of asbestos cause malignant mesothelioma (MM), and cancer of lung, larynx and ovary in humans (Class 1), while the evidence was lower for pharynx, stomach and colorectal cancer (Class 2A) [1]. The evidence of association shows a complex relation between the amount of exposure (dose and duration) and its time pattern (latency and time since cessation) [2] that still deserves clarification from large studies investigating dose, latency and other time related factors.

The asbestos cement industry was the largest asbestos consumer, using 85% of asbestos produced or imported in European countries [3]. It employed a large number of workers: asbestos cement workers in Italy were estimated in 9000 in 1979 and 5000 in 1987, active in a large number of plants, most of which were of small or medium size [4, 5].

Several mortality studies on asbestos cement workers have been conducted in the past decades in Canada [6], United States [7], Israel [8], Italy [9–18], and in other European countries [19–28]. Only few studies analysed the mortality by gender [12, 14, 18, 19, 21, 25, 27, 28], generally including small numbers of female workers and observed deaths. Moreover, some relevant questions, such as the trend of the risk of mesothelial MN with cumulative dose and latency, are still debated [14, 29–31] and are of interest for both preventive and compensation purposes. The results of cohort studies on asbestos cement workers are also of interest for the countries where the product is still made. In all countries, the basic production process is the “Hatschek process”, first patented in late nineteenth century. The information on present asbestos cement industry are scanty and refer of poor working conditions even in recent years [32–35].

The present multicentre investigation is part of a larger project on epidemiological surveillance of asbestos workers [36]. It aimed at investigating mortality by cause, gender, and time-related variables, in particular cumulative dose and latency, with particular attention to the neoplasms with sufficient or limited evidence of association with asbestos [1].

## Material and methods

This study includes 21 asbestos cement factories, part of the Italian pooled cohorts of asbestos workers [36]. The inclusion criteria for individual cohorts were availability of a data set with updated follow-up and a period of observation longer than 40 years. The main characteristics of the cohorts are reported in Additional file 1: Table S1.

**Fig. 1:**
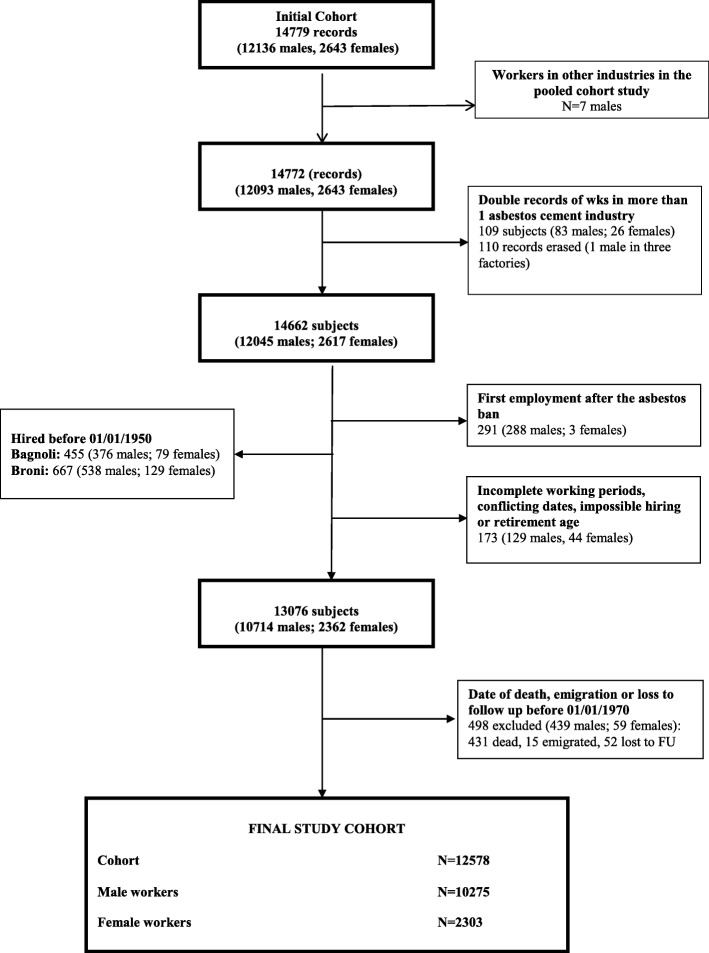
Pooled cohort study of asbestos cement workers in Italy. Flow chart: exclusion criteria and number of subjects included in the analyses

The initial study base included 14,779 workers: 12,136 men (82.1%) and 2643 women (17.9%). During the preliminary analyses 11.5% of records (1703) were excluded, distributed over most of the cohorts, for the following reasons: first employment after 1992 (approval of the asbestos ban in Italy), conflicting dates, incomplete working periods, or unlikely age at hiring or end of employment (Hiring < 13 or > 70; End> 70). The two cohorts of the Eternit-Naples and Fibronit-Broni factories were limited to the workers hired after January 1st, 1950 because the quality of follow-up (FU) data was too limited before that date. Workers employed in more than one plant were identified, and their separate work histories were merged, except for cohort specific analyses. Figure 1 shows the flow chart for the construction of the pooled cohort. The final data set includes 12,578 workers, 81.7% men and 18.3% women.

Vital status was ascertained through the Registrar Office of the municipality of last residence. The FU date was December 31st, 2010 for the three cohorts of Tuscany and the cohort of Lazio, and December 31st, 2012 or later for the other 17 cohorts. For subjects lost to FU, the last contact date was used. Causes of death until 1985 were obtained from the municipalities, afterwards from Local Health Authorities (LHA). Causes were coded according to the 8th, 9th, or 10th Revisions of the International Classification of Diseases (ICD), according to the date of death. The ICD 10th revision is used in Italy from 2003 [37]. Additional file 1: Table S2 presents the causes of death considered and the corresponding codes. We included ‘a priori’ in the analyses the causes of death associated with asbestos exposure, in particular asbestosis, malignant neoplasms (MN) of pleura and peritoneum, larynx, lung, and ovary, and the other neoplasms with limited evidence of association according to IARC classification [1]. Causes of death reported in association with the Healthy Worker Effect (HWE) were considered in the analyses, in particular cardiovascular diseases, digestive diseases, respiratory diseases, psychiatric diseases, neurological diseases, digestive diseases and genitourinary diseases, as well as the total mortality [38]. Cardiovascular diseases and respiratory diseases were also evaluated as related to smoking.

The production cycle of asbestos cement industries in Italy was similar in the different companies and was based on the “Hatschek process” [39]. The dry mixture used for most productions contained about 13–15% of asbestos [39], mostly chrysotile from Italy, Canada and former Soviet Union. Amphibole fibres, mostly crocidolite from Australia and South Africa and a small percentage of amosite from South Africa, were also used depending on the product type. In the production of high-pressure pipes the percentage of asbestos was higher, reaching 20% (usually 1/3 of crocidolite, 1/3 of long chrysotile from Canada, 1/3 Balangero chrysotile). Asbestos exposure was measured in some companies at different times and the results have been reported in scientific papers [10–14, 16, 18], in courts during litigations and in reports from industrial hygiene laboratories of Italian public health agencies or universities. The reported levels of exposure were very high before 1980 (0.2–45 fibres/cc), intermediate between 1980 and 1989 (0.2–11) and lower after 1989 (< 0.1–0.3).

The main products were plain or corrugated sheets, pipes, slabs, tanks, chimneys and other products. In many factories, production also included the manual manufacture of small pieces. The asbestos cement manufacturing process can be reassumed in three stages: mixing, moulding and finishing. Asbestos exposure was high in mixing and finishing while in moulding area, exposure was lower due to the materials being wet. Diffusion of dust from higher to lower exposure areas was common, especially in the plants without physical barriers to separate the areas. These two main modes of exposure have been defined as “direct” for the high exposure areas and “indirect” for the lower ones. Indirect exposure regarded in particular the workers of the moulding workshops, while direct exposure affected the workers employed in mixing and finishing operations.

Two expert industrial hygienists (AA and SS) collected and evaluated the information regarding the use of asbestos, the work process, the plant layout and the measurements of airborne asbestos fibres, for each plant and year of activity. Considered data sources included both published and unpublished reports, in particular company reports, surveys of exposure, judicial examinations, and reports collected from workers [40]. For each factory and calendar period, the original investigators had provided available information, including estimates of the proportion of exposed workers, the percentage of typical working time in tasks with asbestos exposure and the range of minimum and maximum level of asbestos concentration separately for direct and indirect exposures. Company-specific data were checked against those from other factories included in our pooled study and literature data to identify possible inconsistencies (i.e. differences that could not be explained by plant-specific features) and to fill data gaps (e.g. by deriving time-trends for asbestos concentration).

For each plant and periods, the experts estimated the proportion of workers exposed, the percentage of typical working time in tasks with asbestos exposure and the range of minimum and maximum concentration of asbestos airborne fibres (f/ml), separately for direct and indirect exposure.

Tasks and jobs of individual workers were not known, therefore plant and period-specific data were used to compute for each plant and year an Average Exposure Index (AEI) to be applied to all members of a given cohort. First, the ranges of concentration for direct and indirect exposures were summarized in single values by computing the geometric mean between minimum and maximum levels, adjusting for the average proportion of time in tasks with asbestos exposure. Such geometric means were taken as estimates of the time-weighted average level of exposure for direct and indirect exposures, specific for factory and period. The AEI value was calculated for each plant and year as the average of direct and indirect exposures, weighted by the respective proportional size of the workforce, according to the following formula:

$$ AE{I}_{py}=\left({E}_{dpy}\ast {w}_{dpy}+{E}_{ipy}\ast {w}_{ipy}\right) $$

where *E* = Exposure geometric mean, *w* = proportional size of the workforce and *d* = direct, *i* = indirect exposure, and *p* = plant, *y* = year.

From the AEI a Cumulative Average Exposure Index (CEI) was computed for the occupational history of each worker summing the contribution of all periods of activity:

$$ CEI=\sum \limits_{py} AE{I}_{py} $$

Additionally, a fibre-type-weighted-AEI was computed based on the proportion of chrysotile (CH_py_), amosite (A_py_), and crocidolite (CR_py_) used yearly in each plant. The weights were the MM potency factors for chrysotile, amosite and crocidolite (respective 1:14:71) as estimated by Hodgson and Darnton [41]. The fibre-type-weighted-AEI provided the average chrysotile equivalent asbestos concentration in fibres per ml. It was computed as:

$$ fibre- type- weighted\ {AEI}_{py}={AEI}_{py}\ast \left(1\ast {CH}_{py}+14\ast {A}_{py}+71\ast {CR}_{py}\right) $$

A fibre-type-weighted-Cumulative Exposure Index (fibre-type-weighted-CEI) was computed for each worker summing the fibre-type-weighted AEI for all periods of activity.

$$ fibre- type- weighted- CEI=\sum \limits_{py} fibre- type- weighted- AE{I}_{py} $$

On a heuristic ground, we used the same weights also for the analysis of other Asbestos Related Diseases (ARD)s. Sensitivity analyses were conducted using factors derived by other authors [42, 43].

The dimension of AEI was a concentration, expressed in fibres/ml and the dimension of the fibre-type-weighted-AEI was the equivalent concentration of chrysotile asbestos fibres, in fibres/ml. CEI and fibre-type-weighted-CEI had the dimension of concentration times years (f/ml*year), the latter being the equivalent concentration of chrysotile asbestos fibres times years. Additional file 1: Table S3 data presents the exposure indexes by plant and period of activity.

Standardized Mortality Rates (SMR) were stratified by gender, and ‘a priori’ defined classes of calendar time, cumulative exposure, duration and Time Since First Exposure (TSFE) (also known as ‘latency’). For the purpose of present analyses, CEI was categorized in tertiles, based on the distribution of the total cohort. Class limits were: 5.0 and 48.5 f/ml*years for CEI (median 22.0); 54.0 and 620.0 (median 248.0) f/ml*years for fibre-type-weighted-CEI. Duration of exposure was calculated by summing up all the work periods since the date of first employment. TSFE was calculated from the date of first employment until the most recent date of observation. The same categories were used for Poisson regression models.

The numbers of expected deaths (used for SMRs) were based on regional mortality rates provided by the National Institute of Statistics – ISTAT (Rome, Italy) by cause, gender, and year, available from 1970 [37]. Correspondingly, all analyses (including Poisson regression) were restricted to person years (PY) and events occurring after January 1st 1970; subjects lost to follow-up or dead before that date were excluded. For each cohort, expected deaths were calculated with the application of the corresponding regional rates. Multivariable analyses were carried out using Poisson regression [44]. Chi square test for linear trend of SMRs was calculated when appropriate [44]. 95% Confidence Intervals (CI) were computed according to the Poisson distribution of observed deaths [44]. Statistical significance was set at 5%.

Models including restricted cubic splines (4 degrees) on fibre-type-weighted-CEI, TSFE and period were fitted to take into account the possible non linear relation between the predictive variables and mortality [45]. In these models, fibre-type-weighted-CEI and TSFE were analysed as continuous variables.

Data were prepared using MS Access and SAS 9.2. Analyses were carried out using OCMAP plus, STATA 11, SAS 9.2 and R 3.2.5 with Survival and RMS packages [46].

## Results

Table 1 presents the distribution of cohort members (10,275 male and 2303 female workers) and person-years (PYs) (309,675 in men and 79,241 in women), by vital status, year, age at first exposure (defined as the beginning of employment period) and duration of employment. Forty four percent of men and 54.1% of women were alive at the end of FU; 54.4 and 44.9% died, and 1.3 and 0.9% emigrated or were lost at FU. The cause of death was known for 95.9% of male and 97.6% of female decedents. Year of first exposure was before 1970 for 70.2% of workers; 60.1% were younger than 30 at the beginning of activity; duration of employment was shorter than 20 years in 76%. Additional file 1: Table S4 presents the distribution of PY by age class and gender.

**Table 1:** Pooled cohort study of asbestos cement workers in Italy. Descriptive analyses of the cohort

		Males			Females			Total		
		n	%	PY^b^	n	%	PY^b^	n	%	PY^b^
Status at follow-up	alive	4559	44.4	--	1247	54.1	--	5806	46.1	--
	deceased^a^	5591	54.4	--	1035	44.9	--	6626	52.7	--
	emigrated	47	0.5	--	11	0.5	--	58	0.5	--
	lost to follow-up	78	0.8	--	10	0.4	--	88	0.7	--
Year of first exposure	< 1950	974	9.5	18112.3	473	20.5	12727.0	1447	11.5	30839.3
	1950-1959	2124	20.7	59769.5	879	38.2	32382.6	3003	23.9	92152.1
	1960-1969	3870	37.7	130476.4	512	22.2	19699.8	4382	34.8	150176.4
	1970-1979	1831	17.8	62114.4	257	11.2	9247.6	2088	16.6	71362.0
	1980-1989	1352	13.2	36766.5	178	7.7	5096.7	1530	12.2	41863.2
	1990-1992	124	1.2	2435.4	4	0.2	87.1	128	1.0	2522.5
Age at first exposure (years)	< 20	1570	15.3	53787.0	953	41.4	34310.6	2523	20.1	88097.6
	20-29	4255	41.4	138017.6	776	33.7	27258.9	5031	40.0	165276.5
	30-39	2673	26.0	77012.3	388	16.8	12297.4	3061	24.3	89309.6
	40-49	1343	13.1	32209.6	168	7.3	4946.0	1511	12.0	37155.7
	50 +	434	1.2	8648.1	18	0.8	427.9	452	3.6	9076.1
Duration of employment (years)	<10	5439	52.9	183166.7	1330	57.8	49598.8	6769	53.8	232765.4
	10-19	2308	22.5	69308.8	469	20.4	16047.5	2777	22.1	85356.3
	20-29	1884	18.3	45219.6	351	15.2	10035.8	2235	17.8	55255.3
	30 +	644	6.3	11979.5	153	6.6	3558.8	797	6.3	15538.3
	Total	10275	100.0	309674.6	2303	100.0	79240.8	12578	100.0	388915.2

^a^ 255 causes of death unknown (230 males and 25 females, in both sexes 3.8% of decedents); ^b^ person-years computed from 1970

Table 2 presents mortality figures (observed and expected deaths, SMRs and 95% CI), by gender. Overall mortality and mortality for all MN were significantly increased in both sexes. Mortality was significantly higher than expected in both sexes for the MN of the respiratory tract category, and for MN of the lung, pleura, and peritoneum, and also for MN of unspecified site among men. Mortality from ovarian MN was higher than expected even if the excess was not statistically significant. Mortality from MN of larynx, pharynx, stomach, colon and rectum was not significantly higher than expected. Mortality from neoplasms of digestive tract excluding peritoneal MM was very close to expected in both genders (Men: SMR = 1.00; 95% CI: 0.92–1.09; Women: SMR = 1.18; 95% CI: 0.96–1.43). Mortality from asbestosis was extremely high (348 deaths in men - SMR: 507- and 51 in women -SMR: 1023), and determined an excess for the general categories of pneumoconiosis and respiratory diseases. Thirty three deaths were attributed to ‘other pneumoconioses’ (32 in men and 1 in women), including 21 silicoses and 12 ‘unspecified pneumoconioses’; the excess was statistically significant in men. In men, mortality was significantly lower than expected for neurological and cardiovascular diseases. In women for no causes of death mortality was significantly lower than expected. Mortality from unspecified causes (0.1% of total deaths in men and 0.2% in women) was increased in both sexes.

**Table 2:** Pooled cohort study of asbestos cement workers in Italy. Cause specific mortality by gender and selected diseases

	Males					Females				
Cause of death	OBS	EXP	SMR	95% CI		OBS	EXP	SMR	95% CI	
All causes	5591	4599.02	1.23 **	1.19	1.26	1035	771.34	1.34 **	1.26	1.43
Malignant neoplasm (MN)	2342	1589.36	1.47 **	1.41	1.53	414	242.81	1.71 **	1.55	1.88
MN lip, oral cavity and pharynx	29	42.97	0.68 *	0.45	0.97	5	2.56	1.95	0.63	4.56
MN digestive organs (incl peritoneum)	639	544.87	1.17 *	1.08	1.27	130	86.24	1.51 **	1.26	1.79
MN oesophagus	39	31.89	1.22	0.87	1.67	–	1.72	–	–	–
MN stomach	136	139.59	0.97	0.82	1.15	22	16.71	1.32	0.83	1.99
MN small intestine	6	2.81	2.14	0.78	4.65	–	0.47	–	–	–
MN colon	117	102.34	1.14	0.95	1.37	26	20.10	1.29	0.85	1.90
MN rectum	40	45.90	0.87	0.62	1.19	11	7.74	1.42	0.71	2.54
MN of liver and intrahepatic bile ducts	102	103.33	0.99	0.81	1.20	10	11.53	0.87	0.42	1.60
MN peritoneum	102	7.19	14.19 **	11.57	17.23	31	2.05	15.14 **	10.29	21.50
MN respiratory organs	1184	552.55	2.14 **	2.02	2.27	130	25.93	5.01 **	4.19	5.95
MN larynx	50	41.83	1.20	0.89	1.58	2	0.63	3.16	0.38	11.42
MN lung	820	490.25	1.67 **	1.56	1.79	38	22.82	1.67 **	1.18	2.29
MN pleura	305	13.65	22.35 **	19.91	25.00	89	1.85	48.10 **	38.63	59.19
MN uterus						21	14.07	1.49	0.92	2.28
MN ovary						19	12.70	1.50	0.90	2.34
MN prostate	93	96.74	0.96	0.78	1.18					
MN bladder	82	70.79	1.16	0.92	1.44	6	3.50	1.71	0.63	3.73
MN kidney	34	37.90	0.90	0.62	1.25	1	4.08	0.25	0.01	1.37
Leukemia and lymphoma	125	108.04	1.16	0.96	1.38	22	20.52	1.07	0.67	1.62
MN unspecified site	64	40.20	1.59 **	1.23	2.03	8	7.11	1.13	0.49	2.22
Psychiatric diseases	40	38.79	1.03	0.74	1.40	17	12.81	1.33	0.77	2.13
Neurological diseases	60	92.79	0.65 **	0.49	0.83	14	23.26	0.60	0.33	1.01
Cardiovascular diseases	1444	1657.41	0.87 **	0.83	0.92	307	304.88	1.01	0.90	1.13
Respiratory diseases	679	307.83	2.21 **	2.04	2.38	92	37.33	2.47 **	1.99	3.02
Bronchitis, emphysema, asthma	150	127.31	1.18	1.00	1.38	16	10.60	1.51	0.86	2.45
Asbestosis	348	0.69	507.22 **	455.32	563.41	51	0.05	1023.34 **	761.95	1345.52
Other pneumoconioses	32	8.00	4.00 **	2.74	5.65	1	0.04	8.42	0.63	139.30
Digestive diseases	276	281.56	0.98	0.87	1.10	45	40.54	1.11	0.81	1.49
Genitourinary diseases	53	59.76	0.89	0.66	1.16	13	10.39	1.25	0.67	2.14
Accidents and violence	221	234.61	0.94	0.82	1.08	35	28.69	1.22	0.85	1.70
Poorly specified causes	77	29.82	2.58 **	2.04	3.23	24	8.37	2.87 **	1.84	4.27
Unknown causes (in “All causes” only)	230					25				

OBS: observed; EXP: expected; SMR: standardized mortality ratio; CI: confidence interval. * *p* < 0.05; ** *p* < 0.01

Mortality by tertile of cumulative exposure is presented in Table 3. The table also includes the *p*-value of the Chi-square test for linear trend. A statistically significant increasing trend was observed in both sexes for the MN of the pleura and of the peritoneum and for asbestosis, as well as for total mortality and total malignancies. The trend for lung cancer mortality was statistically significant in men, while it was irregular in women. Women showed a statistically significant trend also for ovarian cancer, and the SMR was increased in the upper tertile of cumulative exposure. A negative trend was observed in men for cardiovascular and for accidental deaths. The pattern of mortality from ‘other pneumoconioses’ by tertile of cumulative exposure suggested that at least a proportion of those cases are asbestosis cases: we observed an increasing trend, similar to that observed for the ‘asbestosis’ category but with lower SMRs: I tertile: 1 obs, 1.18 exp., SMR: 0.85 (95% CI: 0.02–4.72); II tertile: 6 obs, 2.46 exp., SMR: 2,44 (95% CI: 0.90–5.31); III tertile: 26 obs, 4.39 exp., SMR: 5.92 (95% CI: 3.87–8.68) (data not tabulated).

**Table 3:** Pooled cohort study of asbestos cement workers in Italy. Mortality by gender and cumulative exposure for selected causes of death

Tertile of the index of cumulative exposure (fibre-type-weighted-Cumulative Exposure Index)							
MALES	I (< 54.0 ff/ml - year)		II (54.0–620.0 ff/ml - year)		III (> 620.0 ff/ml - year)		trend
Cause of death	n	SMR (95% CI)	n	SMR (95% CI)	n	SMR (95% CI)	p
All causes	1038	1.15 (1.08–1.22)	1919	1.16 (1.10–1.21)	2634	1.32 (1.27–1.37)	< 0.001
Malignant neoplasm	425	1.26 (1.14–1.38)	765	1.31 (1.22–1.40)	1152	1.73 (1.63–1.84)	< 0.001
MN stomach	38	1.36 (0.96–1.36)	45	0.89 (0.65–1.19)	53	0.87 (0.65–1.14)	
MN colon	24	1.07 (0.68–1.59)	40	1.08 (0.77–1.47)	53	1.24 (0.93–1.62)	
MN rectum	11	1.18 (0.59–2.12)	13	0.80 (0.43–1.37)	16	0.79 (0.45–1.27)	
MN peritoneum	3	1.83 (0.38–5.35)	24	9.34 (5.98–13.90)	75	25.18 (19.80–31.56)	< 0.001
MN respiratory organs	188	1.61 (1.38–1.85)	375	1.81 (1.63–2.00)	621	2.73 (2.52–2.95)	< 0.001
MN larynx	13	1.65 (0.88–2.82)	19	1.24 (0.75–1.94)	18	0.96 (0.57–1.52)	
MN lung	136	1.30 (1.09–1.54)	251	1.36 (1.19–1.53)	433	2.16 (1.96–2.37)	< 0.001
MN pleura	36	11.27 (7.89–15.60)	100	20.77 (16.90–25.27)	169	29.97 (25.62–34.84)	< 0.001
Respiratory diseases	46	0.93 (0.68–1.25)	210	1.89 (1.64–2.16)	423	2.88 (2.61–3.16)	< 0.001
Bronchitis, emphysema, asthma	20	1.19 (0.73–1.84)	73	1.58 (1.24–1.98)	57	0.89 (0.67–1.15)	
Asbestosis	5	50.03 (16.24–116.76)	75	340.69 (267.98–427.06)	268	732.19 (647.14–825.30)	< 0.001
Cardiovascular diseases	280	0.97 (0.86–1.09)	523	0.87 (0.80–0.95)	641	0.83 (0.77–0.90)	0.04
Digestive diseases	59	1.14 (0.87–1.47)	100	0.95 (0.77–1.15)	117	0.94 (0.78–1.13)	
Accidents and violence	79	1.12 (0.89–1.40)	73	0.90 (0.70–1.13)	69	0.83 (0.65–1.05)	
FEMALES	I		II		III		
Causes of death	n	SMR (95% CI)	n	SMR (95% CI)	n	SMR (95% CI)	
All causes	134	1.24 (1.04–1.47)	328	1.20 (1.08–1.34)	573	1.47 (1.35–1.59)	0.010
Malignant neoplasm	54	1.29 (0.97–1.69)	136	1.47 (1.23–1.74)	224	2.06 (1.80–2.35)	< 0.001
MN stomach	5	2.05 (0.66–4.77)	9	1.53 (0.70–2.91)	8	0.95 (0.41–1.88)	
MN colon	4	1.22 (0.33–3.13)	8	1.08 (0.47–2.13)	14	1.48 (0.81–2.49)	
MN rectum	2	1.71 (0.21–6.19)	2	0.73 (0.09–2.62)	7	1.83 (0.74–3.78)	
MN peritoneum	2	6.16 (0.75–22.25)	3	4.00 (0.82–11.67)	26	26.77 (17.49–39.22)	< 0.001
MN respiratory organs	12	2.38 (1.23–4.16)	45	4.42 (3.22–5.91)	73	6.82 (5.35–8.58)	< 0.001
MN larynx	0	–	1	4.23 (0.11–23.59)	1	3.52 (0.09–19.63)	
MN lung	3	0.66 (0.14–1.93)	22	2.43 (1.52–3.67)	13	1.41 (0.75–2.41)	
MN pleura	8	29.05 (12.54–57.25)	22	32.97 (20.66–49.92)	59	64.99 (49.47–83.83)	0.002
MN ovary	3	1.26 (0.26–3.69	3	0.61 (0.13–1.79)	13	2.40 (1.28–4.10)	
Respiratory diseases	6	1.31 (0.48–2.84)	23	1.84 (1.17–2.76)	63	3.11 (2.39–3.98)	0.006
Bronchitis, emphysema, asthma	2	1.98 (0.24–7.16)	6	1.81 (0.66–3.94)	8	1.28 (0.55–2.51)	
Asbestosis	0	–	10	696.53 (333.99–1280.93)	41	1354.40 (971.93–1837.39)	< 0.001
Cardiovascular diseases	36	1.06 (0.75–1.47)	101	1.02 (0.83–1.23)	170	0.99 (0.85–1.15)	
Digestive diseases	9	1.59 (0.73–3.01)	19	1.24 (0.75–1.93)	17	0.87 (0.51–1.40)	
Accidents and violence	10	2.02 (0.97–3.71)	6	0.60 (0.22–1.31)	19	1.38 (0.83–2.15)	

Fibre-type-weighted-CEI: Index of cumulative exposure adjusted for the type of asbestos used; see text for details. n: observed; SMR: standardized mortality ratio; 95%CI: confidence intervals; p for the chi square test for linear trend reported if < 0.05

Mortality by duration of employment is presented in Additional file 1: Table S5. Results were similar to the trends observed for cumulative exposure and are not discussed in details.

Table 4 shows mortality in relation to TSFE (“latency”). The analysis was limited to the causes selected ‘a priori’ because associated with asbestos exposure, and to some categories related to the HWE. In both genders, ‘All causes’ and ‘All MN’ mortality was lower or equal to expected in the first 19 years of TSFE, and increased afterwards. No case of pleural MN was observed in the first 10 years of TSFE. The trend for pleural MN showed in both sexes increasing SMRs up to the 30–39 years class of TSFE, and a plateau or even a reduction at longer TSFE periods. No deaths were observed for MN of the peritoneum in the first two decades, while SMRs were increased from 20 to 29 years of TSFE in men and 40–49 years in women (*p* < 0.01 in both sexes) with no evidence of plateau. Lung cancer showed a trend similar to pleural MN: mortality was lower than expected in the first 10 years of TSFE, then increased to the highest SMR in the 30–39 year class, followed by a decrease. Laryngeal cancer mortality was below the expected values in the first 4 periods, and increased after 40–49 years of TSFE. Only 2 female cases of laryngeal cancer were observed. Deaths from ovarian cancer were observed only after 20 years of TSFE and the SMR was increased after 50 years of TSFE. There were no deaths from asbestosis in the first 10 years of TSFE in men and in the first 30 years in women. Subsequently, there was a continuous increase over the entire observation period, with SMRs higher in women. Mortality from cardiovascular diseases was lower than expected in all age categories for men but not for women.

**Table 4:** Pooled cohort study of asbestos cement workers in Italy. Cause specific mortality by gender and time since first exposure for selected diseases

TIME SINCE FIRST EXPOSURE												
MALES	0–9		10–19		20–29		30–39		40–49		50+	
Causes of death	n	SMR (95% CI)	n	SMR (95% CI)	n	SMR (95% CI)	n	SMR (95% CI)	n	SMR (95% CI)	n	SMR (95% CI)
All causes	137	0.89 (0.75–1.05)	514	1.05 (0.96–1.14)	1099	1.14** (1.08–1.21)	1601	1.29** (1.22–1.35)	1463	1.31** (1.25–1.38)	777	1.31** (1.22–1.40)
Malignant neoplasm	32	0.69* (0.47–0.97)	173	1.02 (0.87–1.18)	469	1.32** (1.20–1.44)	752	1.63** (1.51–1.75)	660	1.70** (1.58–1.84)	256	1.52** (1.34–1.72)
MN stomach	4	0.72 (0.20–1.85)	16	0.84 (0.48–1.36)	25	0.72 (0.47–1.06)	53	1.36* (1.02–1.78)	30	1.03 (0.70–1.47)	8	0.66 (0.29–1.30)
MN colon	3	1.25 (0.26–3.64)	7	0.77 (0.31–1.58)	19	0.94 (0.57–1.47)	32	1.09 (0.75–1.54)	34	1.21 (0.84–1.70)	22	1.66* (1.04–2.51)
MN rectum	–	–	4	0.82 (0.22–2.10)	3	0.29* (0.06–0.85)	11	0.81 (0.41–1.46)	16	1.45 (0.83–2.36)	6	1.22 (0.45–2.65)
MN peritoneum	–	–	–	–	10	5.77** (2.77–10.61)	23	10.38** (6.58–15.57)	46	31.62** (23.15–42.18)	23	47.13** (29.87–70.71)
MN respiratory organs	16	0.97 (0.55–1.57)	80	1.27* (1.00–1.58)	237	1.80** (1.58–2.04)	410	2.50** (2.26–2.75)	321	2.51** (2.25–2.80)	120	2.45** (2.03–2.93)
MN larynx	1	0.57 (0.01–3.16)	4	0.62 (0.17–1.60)	11	0.96 (0.48–1.71)	9	0.77 (0.35–1.47)	20	2.63** (1.61–4.06)	5	1.73 (0.56–4.03)
MN lung	13	0.92 (0.49–1.57)	62	1.13 (0.87–1.45)	185	1.59 ** (1.37–1.84)	289	1.97** (1.75–2.21)	198	1.72** (1.49–1.98)	73	1.67** (1.31–2.10)
MN pleura	–	–	13	13.12** (6.99–22.44)	39	14.98** (10.65–20.48)	109	27.00** (22.17–32.57)	103	25.41** (20.74–30.82)	41	23.67** (16.98–32.11)
Respiratory diseases	4	0.75 (0.20–1.92)	28	1.25 (0.83–1.81)	98	1.82** (1.48–2.22)	188	2.30** (1.98–2.65)	202	2.31** (2.00–2.65)	159	2.79** (2.38–3.26)
Bronchitis, emphysema, asthma	2	0.85 (0.10–3.09)	7	0.60 (0.24–1.24)	36	1.26 (0.88–1.74)	47	1.19 (0.87–1.58)	35	1.17 (0.81–1.63)	23	1.52 (0.96–2.28)
Asbestosis	–	–	9	241.37** (110.36–458.20)	41	442.77** (317.74–600.67)	94	469.65** (379.53–574.73)	106	491.34** (402.26–594.27)	98	729.36** (592.13–888.86)
Cardiovascular diseases	30	0.73 (0.49–1.04)	131	0.82* (0.69–0.98)	282	0.83** (0.74–0.93)	415	0.91 (0.83–1.00)	368	0.88* (0.80–0.98)	218	0.89 (0.77–1.01)
Digestive diseases	14	0.97 (0.53–1.63)	35	0.76 (0.53–1.06)	73	0.99 (0.78–1.25)	77	1.06 (0.84–1.33)	52	1.01 (0.75–1.32)	25	1.06 (0.69–1.56)
Accidents and violence	35	1.19 (0.83–1.66)	55	1.15 (0.87–1.50)	44	0.78 (0.57–1.04)	38	0.77 (0.55–1.06)	25	0.73 (0.47–1.08)	24	1.38 (0.88–2.05)
FEMALES	0–9		10–19		20–29		30–39		40–49		50+	
Causes of death	n	SMR (95% CI)	n	SMR (95% CI)	n	SMR (95% CI)	n	SMR (95% CI)	n	SMR (95% CI)	n	SMR (95% CI)
All causes	9	1.26 (0.58–2.40)	29	1.06 (0.71–1.52)	83	1.09 (0.87–1.36)	196	1.37** (1.18–1.57)	266	1.26** (1.11–1.42)	452	1.48** (1.35–1.62)
Malignant neoplasm	1	0.33 (0.01–1.84)	17	1.38 (0.80–2.21)	46	1.42* (1.04–1.89)	91	1.67** (1.34–2.05)	115	1.70** (1.41–2.05)	144	1.97** (1.66–2.32)
MN stomach	–	–	3	3.28 (0.68–9.57)	2	0.84 (0.10–3.04)	7	1.82 (0.73–3.76)	5	1.11 (0.36–2.59)	5	1.03 (0.34–2.41)
MN colon	–	–	1	1.23 (0.03–6.85)	1	0.43 (0.01–2.42)	7	1.68 (0.68–3.46)	8	1.40 (0.60–2.75)	9	1.30 (0.60–2.48)
MN rectum	–	–	–	–	–	–	2	1.13 (0.14–4.10)	4	1.89 (0.51–4.83)	5	2.13 (0.69–2.48)
MN peritoneum	–	–	–	–	1	3.34 (0.08–18.60)	1	2.05 (0.05–11.40)	7	10.96** (4.41–22.59)	22	46.17** (28.94–69.91)
MN respiratory organs	–	–	4	4.09* (1.11–10.47)	11	3.61** (1.80–6.46)	36	6.30** (4.41–8.72)	39	5.04** (3.59–6.89)	40	4.85** (3.46–6.60)
MN larynx	–	–	–	–	–	–	1	6.31 (0.16–35.16)	–	–	1	6.25 (0.16–34.80)
MN lung	–	–	2	2.38 (0.29–8.59)	5	1.87 (0.61–4.36)	11	2.18* (1.09–3.90)	10	1.46 (0.70–2.69)	10	1.38 (0.66–2.54)
MN pleura	–	–	2	36.54** (4.42–131.98)	6	35.46** (13.01–77.17)	23	63.45** (40.22–95.20)	29	52.38** (35.08–75.23)	29	41.48** (27.78–59.58)
MN ovary	–	–	–	–	2	1.04 (0.13–3.74)	4	1.27 (0.35–3.25)	4	1.14 (0.31–2.93)	9	2.83* (1.30–5.38)
Respiratory diseases	–	–	–	–	1	0.39 (0.01–2.15)	14	2.46** (1.34–4.12)	19	1.88* (1.13–2.94)	58	3.23** (2.46–4.18)
Bronchitis, emphysema, asthma	–	–	–	–	–	–	6	2.73* (1.00–5.95)	2	0.65 (0.08–2.33)	8	2.05 (0.88–4.04)
Asbestosis	–	–	–	–	–	–	5	727.34** (236.09–1697.31)	11	880.35** (439.46–1575.19)	35	1331.02** (927.11–1851.15)
Cardiovascular diseases	3	2.29 (0.47–6.70)	4	0.60 (0.16–1.55)	11	0.48** (0.24–0.85)	60	1.17 (0.89–1.50)	80	0.95 (0.75–1.18)	149	1.08 (0.91–1.27)
Digestive diseases	–	–	1	0.51 (0.01–2.84)	7	1.34 (0.54–2.78)	10	1.17 (0.56–2.16)	16	1.47 (0.84–2.38)	11	0.82 (0.41–1.46)
Accidents and violence	5	4.41* (1.43–10.30)	4	1.70 (0.46–4.34)	5	1.23 (0.40–2.86)	6	1.11 (0.41–2.41)	6	0.91 (0.33–1.97)	9	0.99 (0.45–1.88)

*OBS* observed, *EXP* expected, *SMR* standardized mortality ratio, *CI* confidence interval. * *p* < 0.05; ** *p* < 0.01

Table 5 presents the results of Poisson regression analyses for pleural and peritoneal malignancies and lung MN. Several models were tested; the presented results correspond to the best fitting models, including different sets of variables according to the different diseases and sexes. All best fitting models included cumulative exposure (fibre-type-weighted-CEI). For pleural MN, the RR increased with cumulative exposure, while with TSFE it showed an increase in the first four decades, followed by a plateau, in both genders. For peritoneal MN, both genders showed a monotonic trend of increasing RR with increasing TSFE and cumulative exposure, albeit the small number of cases showed some irregularities for women. For lung neoplasm, in men RR showed an increase with cumulative exposure and a curvilinear trend for TSFE; for women, the model showed an increase of RR with cumulative exposure only from the lowest to the intermediate categories, with no further increase, and TSFE did not contribute to the model fit.

**Table 5:** Pooled cohort study of asbestos cement workers in Italy. Poisson regression analyses of mortality by gender and cumulative exposure for selected diseases

			RR	95% CI	Other terms in the model
Pleural MN					
Men	Cum.exposure	I tertile	1		age and period
		II tertile	2.41	1.61–3.61	
		III tertile	4.88	3.18–7.48	
	TSFE	0–19 years	1		
		20–29 years	2.08	1.08–4.01	
		30–39 years	4.40	2.26–8.54	
		40–49 years	4.48	2.13–9.42	
Women	Cum.exposure	I tertile	1		age and period
		II tertile	1.33	0.57–3.05	
		III tertile	4.35	1.92–9.84	
	TSFE	0–19 years	1		
		20–29 years	1.79	.35–9.10	
		30–39 years	4.59	0.99–21.36	
		40–49 years	4.80	0.96–24.06	
		50 + years	4.59	0.82–25.73	
Peritoneal MN					
Men	Cum.exposure	I tertile	1		period
		II tertile	5.58	1.66–18.80	
		III tertile	16.62	5.01–55.10	
	TSFE	0–29 years	1		
		30–39 years	4.14	1.86–9.19	
		40–49 years	11.21	4.88–25.76	
		50 + years	14.45	5.67–36.86	
Women	Cum.exposure	I tertile	1		period
		II tertile	0.52	0.09–3.17	
		III tertile	4.25	0.94–19.28	
	TSFE	0–29 years	1		
		30–39 years	1.45	0.09–23.99	
		40–49 years	10.35	1.15–93.36	
		50 + years	37.25	4.13–336.26	
Lung MN					
Men	Cum.exposure	I tertile	1		age and period
		II tertile	1.15	0.92–1.43	
		III tertile	1.82	1.45–2.29	
	TSFE	0–19 years	1		
		20–29 years	1.74	1.31–2.32	
		30–39 years	2.22	1.63–3.01	
		40–49 years	1.76	1.23–2.52	
		50 + years	1.34	0.87–2.07	
Women	Cum.exposure	I tertile	1		age
		II tertile	3.75	1.12–12.61	
		III tertile	2.25	0.63–8.07	

Cumulative exposure expressed as Fibre-type-weighted-CEI (Index of cumulative exposure adjusted for the type of asbestos used), see text for details

**Fig. 2:**
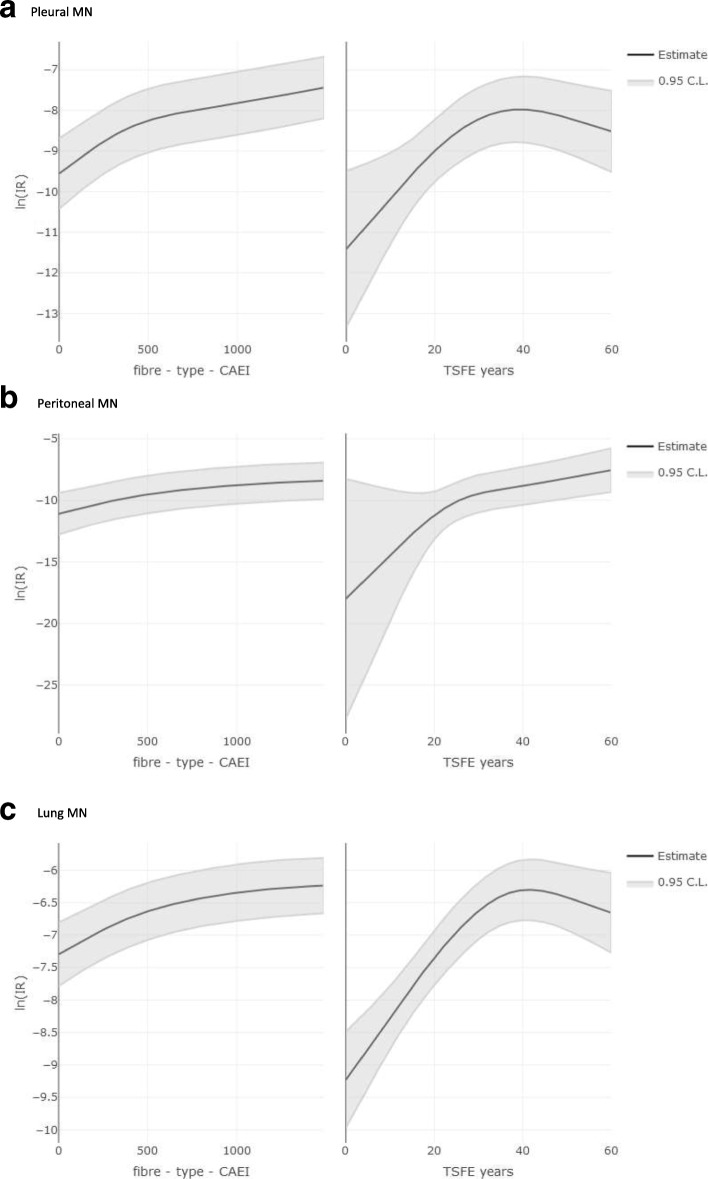
Pooled cohort study of asbestos cement workers in Italy. Restricted cubic spline (4 degrees) for pleural (**a**), peritoneal (**b**), and lung (**c**) MN mortality on Fibre-type-Cumulative Exposure Index (fibre-type-weighted CEI) and Time Since First Exposure (TSFE). The analyses were adjusted for fibre-type-weighted-CEI, TSFE and period. The plots are trimmed at the 90° percentile of the cumulative distribution of the fibre-type-weighted-CEI

Figure 2 presents the cubic spline describing the relation of mortality rate from pleural (a), peritoneal (b) and lung MN (c) with cumulative exposure (fibre-type-weighted-CEI) and TSFE. For pleural neoplasm, mortality continuously increased with cumulative exposure, while it reached a plateau after 30 years of TSFE. Similar trends were observed for lung cancer, with a plateau becoming apparent after 40 years since first exposure. On the opposite, an increasing trend of mortality for peritoneal cancer was observed along the all range of fibre-type-weighted-CEI and TSFE.

Analyses for cumulative exposure were repeated using both the unweighted CEI and the fibre-type-weighted-CEI computed using the weights estimated by Garabrant et al. [42]. None of these analyses (not in details) showed relevant differences with the analyses presented in Tables 3 and 5 and in Fig. 2 and all confirmed the dose response trends with increasing exposure.

Analyses by period of first exposure are reported in Additional file 1: Table S6. Among workers with first employment in 1980–89, a statistically significant excess was observed for pleural MN and for asbestosis (1 case) among men (employed in 1980, aged 36, until 1994), and one case of MN of the pleura was observed in women.

## Discussion

Our study is a pooled analysis of 21 cohorts of asbestos cement workers, which accounted for a wide fraction of workers employed in the asbestos cement production in Italy [4]. At our knowledge, this is the largest study in the world on asbestos cement workers. Other specific features of this study include a very long follow-up, more than 40 years, and a significant number of women, providing a substantial contribution to gender-specific analyses.

The follow-up results are satisfactory, with only 1.3% of subjects with unknown status (lost or emigrated). Causes of death are known for over 95% of decedents in both sexes. SMR analyses are based on regional mortality rates, in order to increase the comparability between cohorts and reference rates. The decision to restrict the analyses to 1970 onwards depended on the availability of reference mortality rates [37] and not on the quality of cohort data, which is overall high. For consistency of the design and analyses, we decided to apply the restriction also to the internal analyses with Poisson regression, even if it was not strictly necessary.

Asbestos exposure could not be assessed on an individual basis, because of the lack of individual data on jobs and work activities for members of almost all cohorts. Therefore, we based our exposure assessment on the average index (AEI) representing the plant and period workforce average exposure, obtained from the estimation of exposure for workers with direct and indirect asbestos exposure. We calculated the individual cumulative exposure index (CEI) of cohort members by applying the AEI specific for plant and period to their duration and timing of employment. To take into account differences in the use of amphibole and chrysotile asbestos by plant and period, indices were weighted for the proportion of the different fibre types and their estimated carcinogenic potency factor for pleural MM [41]. More recent estimates of fibre type potency were used in sensitivity analyses [42], although the data base used for that review was criticized for incompleteness [43], and no relevant difference was observed.

The study was based on mortality data, as cancer registries in Italy do not cover the population and period of interest for the study. There was no specific code for MM of peritoneum and pleura in the 8th and 9th ICD, and ICD 10 ^th^ is in use from 2003 in Italy. The use of mortality data could have caused a misclassification of MM with other cancers, in particular metastasis or lung cancer, in both directions. Therefore, literature evidence was searched to evaluate this possible bias. The sensitivity of death certificates for the identification of MM was explored in a meta-analysis by Kopylev et al. [47], who observed an underestimation of MM incidence from mortality data. This observation was supported by other studies not included in that revision: 74,5% of pleural MM cases could be identified from mortality records in Italy [48] and 87% in Southern England [49]. Similar results were observed by Conti et al., who compared mortality and incidence for peritoneal MM in Italy [50]. Some studies in our cohort [12, 14, 16] had performed a record linkage with the Italian Mesothelioma Registry data: results were satisfactory and showed that SMRs did not over-estimate SIRs of MM.

Mortality for “All causes” and “All MN” showed in both sexes a statistically significant increase, in general and in stratified analyses, in particular according to cumulative exposure and TSFE. The pattern was observed also for the workers who started employment in the more recent periods. This overall result, which is not affected by questions regarding classification of causes of deaths, shows macroscopically the consequences of the exposure in the asbestos-cement production industries. In total, the cohorts in study showed an excess of 1255.6 deaths, corresponding to a 19% increase of overall mortality. This large excess should be evaluated also with consideration of the evidence of a relevant HWE, as shown by the low mortality for cardiovascular and neurological diseases and by the low mortality observed in the first ten years of TSFE.

A statistically significant increase in mortality was observed in both sexes for pleural, peritoneal, and lung MN, consistent with the recent IARC evaluation [1]: overall the number of deaths were 394, 133 and 858, and the corresponding attributable proportions [51] for these conditions were respectively 96, 93 and 40%, similar for men and women. The point estimate of SMRs in women are higher than in men for peritoneal and pleural MN. These results do not reflect a higher risk or an increased sensitivity to asbestos, but rather denote the lower female reference mortality rates in general population [37].

Analyses by cumulative exposure showed a statistically significant trend for pleural and peritoneal MN and for lung cancer, providing additional support to the evidence of dose response relation for MM [52] and for lung cancer [53, 54]. Poisson regression analyses showed similar RRs in men and women for pleural MN. For peritoneal MN, men had higher RRs than women at the same levels of cumulative exposure, however the number of female cases was limited and confidence intervals were wide and overlapping between the two group.

In our study, SMRs for both pleural and lung MN increased up to 30–39 years of TSFE and showed a plateau thereafter, with some fluctuations in women. On the contrary, the SMR for peritoneal MN increased monotonically in both genders. The same trends were observed also in the Poisson regression analyses using splines. As regards MM, our results contradict the traditional models of asbestos carcinogenesis, that predicted an unlimited increase of MM rates over time from the beginning of asbestos exposure, according to a power function of TSFE [55, 56] and did not differentiate between the anatomical location of MM [55, 56]. On the contrary, our results supported the evidence of a flattening in the trend of MM rates with TSFE and showed a difference between pleural and peritoneal MN, in agreement with the more recent evidence showing a reduction of the increasing incidence trend after long latency for pleural but not for peritoneal MM [14, 29–31, 57, 58]. The only exception in the recent literature was a follow-up study of former Polish asbestos workers participating in a health surveillance program, among whom a continued increase in the risk of pleural MM by TSFE was reported [59]. The interpretation of such study is difficult as results may have been biased by the different participation of subjects with symptoms or otherwise interested in health assessment. A biological interpretation of the evidence is that asbestos fibres are slowly cleared from the lungs. For amphibole asbestos, a constant annual elimination rate in the order of up to 10% was estimated in different studies [60–66]. Toxicological [67] and histological studies [66] showed a different clearance of the fibres, especially in subjects with chrysotile only exposure. The same explanation holds also for lung cancer, showing results consistent with the reduction of risk following a reduction of lung burden. The difference between pleura and peritoneum is also consistent with reports from other studies [14, 29, 30, 68], and can be explained by the different distribution of asbestos fibers in the body, that interest the pleura more directly and the peritoneum only after internal transportation [69].

Ovarian cancer overall showed a modest increase in mortality, with SMR similar to the RR measured in meta-analyses [70, 71] and in a preliminary analysis of the present cohorts, including other industrial sectors as well as the asbestos cement [36]. However, we observed a significant increase in women in the III tertile of cumulative exposure, in those who started employment before 1950, after more than 30 years of employment, or TSFE longer than 50 years.

We observed only a slight increase of laryngeal cancer mortality in both sexes. In men, the SMRs were higher in the higher classes of TSFE (40–49 and 50+ years) but we did not see a relation with cumulative exposure. In women, the two observed cases were in the mid and upper tertile of cumulative exposure and in the upper categories of TSFE. In summary, our cohort results provide little support to the association with laryngeal cancer, contrary to the two recent reviews by the Institute of Medicine [72] and by Peng et al. [73]. Our study was limited by the use of mortality data and the absence of information on smoking and drinking. Mortality analyses are not very sensitive for diseases with long survival, such as laryngeal cancer in recent periods [74]. A possible (negative) confounding by smoking and drinking habits cannot be ruled out: the mortality analyses showed a reduced mortality for cardiovascular deaths, suggesting that smoking habits were not worse than in the general population, although the evidence in this respect is limited.

Overall this large cohort did not show an excess of MN of digestive organs other than the peritoneal MN and provide little support to the causality of asbestos exposure for these neoplasms, contrary to recent results from meta-analyses or other cohorts [72, 75]. However, it should be noticed that for colon and rectum MN an excess was shown after TSFE longer than 50 years. As discussed for larynx cancer, these tumours have long survival and mortality could not be a sensitive indicator of risk.

For asbestosis, we observed in both sexes a clear association with cumulative exposure, a result that corroborates on the quality of our exposure assessment. A specific work on asbestosis in this cohort study is in progress, however present analyses already provide some relevant results. The increase in mortality from asbestosis was large, in particular among women. We believe that these data do not express a real gender difference but reflect the very low female reference mortality rates from these diseases in Italy [37]. Five cases of death attributed to asbestosis were observed in men in the lowest tertile of cumulative exposure, corresponding to the cumulative exposure ‘up to 54.0’ f/ml*years for fibre-type-weighted-CEI. Mortality from asbestosis by period of first employment declined but remained higher than expected until the most recent periods, with one case observed among workers who started activity after 1980, possibly suggesting high exposures also in the last periods of industrial use of asbestos in Italy. Mortality from asbestosis was assessed on the basis of the underlying cause of death and no best-evidence assignment of the cause of death has been attempted. Previous reports from some of the cohorts included in the present study [14, 16], showed that additional cases had been reported when concomitant causes of death were considered, therefore an underestimation of SMRs is possible in this study. With consideration to the possible misclassification of outcome, we underline that 32 deaths in men and 1 in women had been recorded as “other pneumoconiosis” in the death certificate, possibly attributable to incorrect reporting of asbestosis, as SMRs increased with increasing cumulative exposure.

The possible occurrence of HWE was analysed with consideration of total mortality and of the causes of death reported in the literature as most often associated with it [38], namely cardiovascular, respiratory, neurological, psychiatric, digestive and genitourinary diseases. Overall mortality was higher than expected, because of the large increase of ARD mortality. The pattern of specific causes of death showed a marked HWE effect; in particular results for cardiovascular diseases mortality were corresponding to a HWE from selection at hire and possibly an additionally healthy worker survivor effect [38]: cardiovascular disease mortality was not associated with cumulative exposure and remained lower than expected in the analyses by TSFE.

We do not have any individual information on smoking habits for the study, as often happens in occupational cohort investigations. However, the risk for cardiovascular diseases, strongly associated to smoking, is decreased in the cohort even after long TSFE, suggesting that the proportion of smokers was at least not larger than in the general population. Our findings on lung cancer are, therefore, unlikely due to uncontrolled confounding from smoking. We were, however, unable to assess the combined effect of tobacco smoking and asbestos [54, 76].

## Conclusions

Our study contributes to the evidence of increased mortality risk due to asbestos exposure for MN of pleura, peritoneum, lung and ovary, as well as for asbestosis, all increasing with cumulative exposure. We have not detected an excess risk of larynx, pharynx, and stomach MN, except for larynx cancer after very long latencies. In summary, ARD mortality was higher than expected in both genders. Mortality from cardiovascular diseases was significantly lower than expected in men, suggesting an HWE but also that the prevalence of smokers among asbestos-cement workers was not larger than in the general population. Mortality for smoking-related neoplasms different from lung and larynx cancer was not increased.

Pleural and peritoneal MN showed a clear exposure-response relation and had different trends with TSFE. The TSFE analysis showed a monotonic increase of mortality in relation to TSFE for peritoneal MN, while pleura and lung MN mortality reached a plateau about 40 years after first exposure. The increase of MM rates by TSFE predicted by traditional models might be attenuated, due to the effect of the clearance of fibres.

These results underline the increased risk of ARDs in the asbestos cement production and are therefore of special relevance also for the countries that have not banned the use of asbestos.

## Additional file

Additional file 1: **Table S1.** Asbestos cement pooled Italian study. Summary of cohort data, including all subjects, including those with follow-up terminated before 1970. **Table S2.** Asbestos cement pooled Italian study. Cause of death codes according to ICD in use at the date of death. **Table S3.** Asbestos cement pooled Italian study. Average asbestos exposure index, crude and adjusted for carcinogenic potency of asbestos types used, by cohort. **Table S4.** Asbestos cement pooled Italian study. Person-years at risk by gender and age class. **Table S5.** Asbestos cement pooled Italian study. Mortality by gender and duration of employment for selected causes of death. **Table S6.** Asbestos cement pooled Italian study. Mortality by gender and period of first exposure for selected causes of death. (PDF 646 kb)

## Data Availability

The datasets used and analysed during the current study are available from the corresponding author on reasonable request.

## Abbreviations

AEI: Average Exposure Index
ARD: Asbestos Related Disease
CEI: Cumulative Average Exposure Index
CI: Confidence Interval
FU: Follow-up
HWE: Healthy Worker Effect
IARC: International Agency for Research on Cancer
ICD: International Classification of Diseases
LHA: Local Health Authority
MM: Malignant mesothelioma
MN: Malignant neoplasm
SMR: Standardized Mortality Rate
TSFE: Time Since First Exposure
