# Supplementary material for: Cumulative asbestos exposure and mortality from asbestos related diseases in a pooled analysis of 21 asbestos cement cohorts in Italy
Source: Environ Health. 2019 Aug 7;18:71. doi: 10.1186/s12940-019-0510-6 (PMC6686495; doi:10.1186/s12940-019-0510-6)
Supplement: Supplementary file 1 — Table S1. Asbestos cement pooled Italian study. Summary of cohort data, including all subjects, including those with follow-up terminated before 1970. Table S2. Asbestos cement pooled Italian study. Cause of death codes according to ICD in use at the date of death. Table S3. Asbestos cement pooled Italian study. Average asbestos exposure index, crude and adjusted for carcinogenic potency of asbestos types used, by cohort. Table S4. Asbestos cement pooled Italian study. Person-years at risk by gender and age class. Table S5. Asbestos cement pooled Italian study. Mortality by gender and duration of employment for selected causes of death. Table S6. Asbestos cement pooled Italian study. Mortality by gender and period of first exposure for selected causes of death. (PDF 646 kb) [file 12940_2019_510_MOESM1_ESM.pdf]

Company or cohort name	Town	Region	Period of activity	Asbestos type used	Asbestos used from	Males	Females	References
Fibronit	Bari	Apulia	1934 - 1989	Chrysotile, crocidolite, amosite	1934	413	-	Coviello 2002
Eternit	Naples	Campania	1939 - 1986 #	Chrysotile, crocidolite	1939	1225	222	Menegozzo 2011
Artclit	Cadelbosco di Sopra	Emilia-Romagna	1965 - 1987	Chrysotile, crocidolite	1965	49	1	Luberto 2004
Cemental	Correggio	Emilia-Romagna	1952 - 1989	Chrysotile, crocidolite	1952	473	75	Luberto 2004
Cemiant	Cadelbosco di Sopra	Emilia-Romagna	1968 - 1991	Chrysotile	1968	22	81	Luberto 2004
Eternit / ICAR	Rubiera	Emilia-Romagna	1961 - 1992	Chrysotile, crocidolite	1961	484	69	Luberto 2004
Fibrotubi	Bagnolo in Piano	Emilia-Romagna	1957 - 1993	Chrysotile, crocidolite	1957	230	54	Luberto 2004
Itamiant	Castelnovo di Sotto	Emilia-Romagna	1955 - 1993	Chrysotile, crocidolite	1955	884	303	Luberto 2004
Maranit	Poggio Renatico	Emilia-Romagna	1962 - 1993	Chrysotile, crocidolite	1962	184	17	Luberto 2004
Sidercam	Boretto	Emilia-Romagna	1969 - 1993	Chrysotile, crocidolite	1969	117	11	Luberto 2004
Superlit	Novi di Modena	Emilia-Romagna	1954 - 1993	Chrysotile, crocidolite	1954	169	6	Luberto 2004
Uprocem	Boretto	Emilia-Romagna	1973 - 1993	Chrysotile, crocidolite	1973	43	15	Luberto 2004
Cemamit	Ferentino	Lazio	1963 - 1984	Chrysotile, crocidolite	1963	80	1	-
Fibronit	Broni	Lombardy	1932 - 1993 #	Chrysotile, crocidolite, amosite	1932	1294	39	Oddone 2017
Sacelit	Senigallia	Marche	1948 - 1984	Chrysotile, crocidolite, amosite	1948	450	139	Pettinari 1994
SACA	Cavagnolo	Piedmont	1948 - 1982	Chrysotile, crocidolite, amosite	1948	547	265	Ferrante 2008
Eternit	Casale Monferrato	Piedmont	1907 - 1986	Chrysotile, crocidolite	1907	2608	758	Magnani 2008
Baraclit	Bibbiena	Tuscany	1943 - active	Chrysotile from 1945; Mixed from 1963	1945	725	-	-
Fibronit	Avenza	Tuscany	1940 - 1983	Mixed fibres	1935	189	37	Raffaelli 2007
Veronit	Livorno	Tuscany	1930 - 1985	Mixed fibres	1950	128	29	-
Edilit	Vigodarzere	Veneto	1961 - active	Chrysotile, crocidolite	1947	323	216	-
Workers in multiple cohorts						77	24	
**Total**						**10714**	**2362**	

Supplementary table 1. Asbestos cement pooled Italian study. Summary of cohort data, including all subjects, including those with follow-up terminated before 1970.
Restricted to workers first employed from 1950

	VIII	IX	X
All causes	0000-9999	0001-9999	A00-T98
Malignant neoplasm (MN)	1400-2079	1400-2089	C00-C97
MN of the lip, oral cavity and pharynx	1400-1499	1400-1499	C00-C14
MN of the digestive organs (incl peritoneum)	1500-1599, 1978-1978	1500-1599	C15-C26,C45.1,C48
MN of the stomach	1510-1519	1510-1519	C16
MN of the small intestine	1520-1529	1520-1529	C17
MN of the colon	1530-1539	1530-1539	C18
MN of the rectum	1540-1549	1540-1549	C19-C21
MN of the liver and intrahepatic bile ducts	1550-1559, 1978-1978	1550-1552	C22
MN of the peritoneum	1580-1589	1580-1589	C48,C45.1
MN of the respiratory organs	1600-1639, 1942-1942	1600-1659	C30-C39, C45.0, C45.2, C45.9
MN of the larynx	1610-1619	1610-1619	C32
MN of the lungs	1620-1629	1620-1629	C33-C34
MN of the pleura	1630-1630	1630-1639	C38.4, C45.0, C45.9
MN of the uterus	1800-1809, 1820-1829	1790-1809, 1820-1829	C53-C55
MN of the ovaries	1830-1839	1830-1839	C56-C57
MN of the prostate	1850-1859	1850-1859	C61
MN of the bladder	1880-1889	1880-1889	C67
MN of the kidney	1890-1899	1890-1899	C64-C66,C68
Leukaemia and lymphoma	2000-2079	2000-2089	C81-C96
MN of unspecified site	1990-1999	1990-1999	C80
Psychiatric diseases	2900-3199	2900-3199	F00-F99
Neurological diseases	3200-3589, 0660-0669	3200-3599	G00-G99
Cardiovascular diseases	3900-4589	3900-4599	I00-I26,I28-I99
Respiratory diseases	4600-5199	4600-5199	J00-J99
Digestive diseases	5200-5799	5200-5799	K00-K93
Genitourinary diseases	5800-6299	5800-6299	N00-N99
Asbestosis	5152-5152	5010-5019	J61
Pneumoconiosis	5150-5169	5000 - 5059	J60- J65
Accidents and violence	8000-9999	8000-9999	S00-T98
Poorly specified causes	7800-7963	7800-7998	R00-R98

Supplementary table 2. Asbestos cement pooled Italian study. Cause of death codes according to ICD in use at the date of death.

Plant	Period	AEI index (f/ml)	Fibre-type-weighted-AEI (chrysotile equivalent f/ml)
Fibronit - Bari	1934 - 1974	3.9	55.9
Fibronit - Bari	1975 - 1980	0.6	55.9
Fibronit - Bari	1981 - 1985	0.4	8.6
Fibronit - Bari	1986 - 1989	0.0	5.3
Eternit - Naples	1950 - 1974	3.6	29.5
Eternit - Naples	1975 - 1986	0.9	7.7
Eternit - Naples	1987 - 1988	0.0	0.0
Artclit - Cadelbosco di S.	1965 - 1974	3.6	131.3
Artclit - Cadelbosco di S.	1975 - 1983	0.4	13.4
Artclit - Cadelbosco di S.	1984 - 1988	0.2	0.2
Cemental - Correggio	1952 - 1974	3.6	80.4
Cemental - Correggio	1975 - 1979	1.6	23.9
Cemental - Correggio	1980 - 1987	0.5	7.9
Cemental - Correggio	1988 - 1989	0.3	0.2
Cemiant - Cadelbosco di S.	1968 - 1974	3.6	3.6
Cemiant - Cadelbosco di S.	1975 - 1982	1.6	1.6
Cemiant - Cadelbosco di S.	1983 - 1986	0.3	0.3
Cemiant - Cadelbosco di S.	1987 - 1991	0.0	0.0
Eternit/ICAR - Rubiera	1961 - 1975	5.6	124.8
Eternit/ICAR - Rubiera	1976 - 1979	0.9	20.9
Eternit/ICAR - Rubiera	1980 - 1992	0.1	0.1
Fibrotubi - Bagnolo in P.	1957 - 1974	3.9	31.5
Fibrotubi - Bagnolo in P.	1975 - 1979	1.3	10.7
Fibrotubi - Bagnolo in P.	1980 - 1983	0.3	2.4
Fibrotubi - Bagnolo in P.	1984 - 1993	0.0	0.0
Itamiant - Castelnovo di S.	1955 - 1974	5.0	109.7
Itamiant - Castelnovo di S.	1975 - 1979	1.3	29.2
Itamiant - Castelnovo di S.	1980 - 1986	0.1	2.7
Itamiant - Castelnovo di S.	1987 - 1993	0.0	0.1
Maranit - Poggio Renatico	1962 - 1974	5.0	75.0
Maranit - Poggio Renatico	1975 - 1979	1.0	15.4
Maranit - Poggio Renatico	1980 - 1985	0.3	3.9
Maranit - Poggio Renatico	1986 - 1993	0.0	0.0
Sidercam - Boretto	1969 - 1974	5.0	22.9
Sidercam - Boretto	1975 - 1985	0.5	2.4
Sidercam - Boretto	1986 - 1993	0.0	0.0
Superlit - Novi di Modena	1954 - 1974	3.9	45.1
Superlit - Novi di Modena	1975 - 1986	0.5	6.1
Superlit - Novi di Modena	1987 - 1993	0.0	0.0
Uprocem - Boretto	1973 - 1974	5.0	57.6
Uprocem - Boretto	1975 - 1982	1.8	20.8
Uprocem - Boretto	1983 - 1984	0.5	6.1
Uprocem - Boretto	1985 - 1986	0.1	1.2
Uprocem - Boretto	1987 - 1993	0.0	0.0
Cemamit - Ferentino	1963 - 1974	3.6	29.5
Cemamit - Ferentino	1975 - 1976	1.6	12.8
Cemamit - Ferentino	1977 - 1984	0.5	4.2
Fibronit - Broni	1932 - 1974	3.6	52.4
Fibronit - Broni	1975 - 1980	0.3	4.3
Fibronit - Broni	1981 - 1986	0.4	5.3
Fibronit - Broni	1987 - 1990	0.2	0.4
Fibronit - Broni	1991 - 1993	0.1	0.1
Fibronit - Broni	1994 - 1997	0.0	0.0
Sacelit - Senigallia	1948 - 1974	3.9	31.5
Sacelit - Senigallia	1975 - 1977	1.0	8.1
Sacelit - Senigallia	1978 - 1984	0.3	2.1
SACA - Cavagnolo	1947 - 1960	5.0	109.7
SACA - Cavagnolo	1961 - 1975	3.9	59.1
SACA - Cavagnolo	1976 - 1978	0.5	4.2

SACA - Cavagnolo	1979 - 1982	0.3	2.1
SACA - Cavagnolo	1983 - 1983	0.0	0.0
Eternit - Casale Monferrato	1907 - 1974	5.5	78.7
Eternit - Casale Monferrato	1975 - 1986	0.3	4.3
Baraclit - Bibbiena	1943 - 1959	2.1	2.1
Baraclit - Bibbiena	1960 - 1974	0.8	1.4
Baraclit - Bibbiena	1975 - 1980	0.2	0.4
Baraclit - Bibbiena	1981 - 1984	0.0	0.1
Baraclit - Bibbiena	1985 - 2001	0.0	0.0
Fibronit - Avenza	1935 - 1974	5.0	40.3
Fibronit - Avenza	1975 - 1978	4.3	34.9
Fibronit - Avenza	1979 - 1985	1.8	14.6
Fibronit - Avenza	1986 - 1988	0.0	0.0
Veronit - Livorno	1950 - 1961	6.2	50.1
Veronit - Livorno	1962 - 1979	6.1	49.5
Veronit - Livorno	1980 - 1989	2.0	16.1
Edilit - Vigodarzere	1946 - 1974	3.6	22.9
Edilit - Vigodarzere	1975 - 1984	0.9	5.9
Edilit - Vigodarzere	1985 - 1994	0.0	0.2
Edilit - Vigodarzere	1995 - 2006	0.0	0.0

Supplementary table 3. Asbestos cement pooled Italian study. Average asbestos exposure index, crude and adjusted for carcinogenic potency of asbestos types used. See text.

Age class	Males	Females	Total
10-19	1472	274	1746
20-29	19006	3392	22398
30-39	43011	9779	52790
40-49	67871	16424	84295
50-59	75232	18312	93544
60-69	60825	16916	77741
70-79	33475	10768	44243
80-89	8273	3126	11399
90+	509	250	759
**Total**	**309674**	**79241**	**388915**

Supplementary table 4. Asbestos cement pooled Italian study. Person-years at risk by gender and age class.

OBS: observed; EXP: expected; SMR: standardized mortality ratio; CI: confidence interval. * p < 0.05; ** p < 0.01.

	Duration of employment							
**MALES**	**0-9**		**10-19**		**20-29**		**30+**	
**Cause of death**	**n**	**SMR (95% CI)**	**n**	**SMR (95% CI)**	**n**	**SMR (95% CI)**	**n**	**SMR (95% CI)**
All causes	2268	1.16** (1.11-1.21)	1499	1.25** (1.18-1.31)	1347	1.30** (1.24-1.38)	477	1.31** (1.19-1.43)
Malignant neoplasm	910	1.30**(1.22-1.39)	626	1.52** (1.40-1.64)	607	1.68** (1.54-1.81)	199	1.71** (1.48-1.97)
MN stomach	68	1.13 (0.88-1.43)	32	0.85 (0.58-1.20)	26	0.83 (0.54-1.22)	10	0.96 (0.46-1.77)
MN colon	46	1.01 (0.74-1.34)	39	1.51* (1.07-2.06)	20	0.86 (0.53-1.33)	12	1.60 (0.83-2.80)
MN rectum	17	0.86 (0.50-1.37)	9	0.76 (0.35-1.44)	13	1.24 (0.66-2.12)	1	0.27 (0.01-1.52)
MN peritoneum retroperitoneum	22	6.66** (4.18-10.09)	24	13.12** (8.41-19.52)	36	23.06** (16.15-31.93)	20	40.48** (24.73-62.51)
MN respiratory organs	436	1.80** (1.65-1.98)	312	2.16** (1.93-2.42)	334	2.64** (2.36-2.94)	102	2.59** (2.11-3.15)
MN larynx	27	1.55* (1.02-2.26)	9	0.77 (0.35-1.47)	12	1.24 (0.64-2.17)	2	0.64 (0.08-2.32)
MN lung	307	1.42** (1.27-1.59)	229	1.80** (1.57-2.04)	217	1.93** (1.68-2.21)	67	1.92** (1.49-2.44)
MN pleura	97	15.45** (12.53-18.85)	71	21.74** (16.98-27.43)	104	32.87** (26.86-39.83)	33	35.06** (24.13-49.23)
Respiratory diseases	169	1.40* (1.19-1.62)	197	2.34** (2.02-2.69)	221	3.00** (2.62-3.42)	92	3.19** (2.57-3.91)
Bronchitis, emphysema, asthma	71	1.54** (1.20-1.94)	39	1.05 (0.74-1.43)	35	1.15 (0.80-1.61)	5	0.37* (0.12-0.86)
Asbestosis	35	135.60** (94.45-188.59)	105	635.35** (519.64-769.14)	134	720.32** (630.52-853.13)	74	964.89** (757.65-1211.33)
Cardiovascular diseases	635	0.94 (0.86-1.01)	375	0.84** (0.76-0.93)	314	0.82** (0.73-0.91)	120	0.82* (0.68-0.98)
Digestive diseases	122	1.03 (0.86- 1.23)	81	1.03 (0.82-1.28)	56	0.89 (0.677-1.16)	17	0.78* (0.46-1.25)
Accidents and violence	133	1.07 (0.90-1.27)	45	0.80 (0.58-1.07)	26	0.63* (0.41-0.92)	17	1.31 (0.76-2.10)
**FEMALES**	**0-9**		**10-19**		**20-29**		**30+**	
**Causes of death**	**n**	**SMR (95% CI)**	**n**	**SMR (95% CI)**	**n**	**SMR (95% CI)**	**n**	**SMR (95% CI)**
All causes	428	1.22** (1.10-1.34)	265	1.34** (1.18-1.51)	223	1.46** (1.28-1.67)	119	1.74** (1.44-2.08)
Malignant neoplasm	171	1.39** (1.19-1.61)	105	1.80** (1.47-2.18)	84	1.97** (1.57-2.43)	54	2.89** (2.17-3.77)
MN stomach	13	1.71 (0.91-2.92)	3	0.70 (0.15-2.05)	5	1.49 (0.48-3.47)	1	0.69 (0.02-3.86)
MN colon	11	1.11 (0.56-1.99)	8	1.64 (0.71-3.22)	6	1.62 (0.59-3.52)	1	0.61 (0.02-3.42)
MN rectum	3	0.82 (0.17-2.40)	6	3.14* (1.15-6.84)	1	0.67 (0.02-3.74)	1	1.44 (0.04-8.01)
MN peritoneum retroperitoneum	3	3.01 (0.62-8.81)	13	25.77** (13.72-44.06)	9	23.84** (10.90-45.25)	6	35.39** (12.99-77.02)
MN respiratory organs	50	3.59** (2.67-4.74)	31	5.15** (3.50-7.32)	28	6.65** (4.42-9.62)	21	11.68** (7.23-17.85)
MN larynx	1	3.14 (0.08-17.47)	-	-	1	9.13 (0.23-50.90)	-	-
MN lung	21	1.70* (1.05-2.60)	8	1.52 (0.66-2.99)	3	0.82 (0.17-2.40)	6	3.90* (1.43-8.49)
MN pleura	27	29.33** (19.33-42.67)	23	53.14** (33.69-79.74)	24	71.32** (45.70-106.12)	15	93.49** (52.32-154.19)
MN ovary	6	0.89 (0.33-1.94)	5	1.71 (0.55-3.98)	4	1.89 (0.52-4.83)	4	4.38* (1.19-11.21)
Respiratory diseases	28	1.73** (1.15-2.50)	23	2.31** (1.46-3.47)	26	3.38** (2.21-4.95)	15	4.27** (2.39-7.05)
Bronchitis, emphysema, asthma	9	2.24* (1.02-4.25)	4	1.36 (0.37-3.48)	-	-	3	2.53 (0.52-7.41)
Asbestosis	9	414.39** (189.47-786.64)	16	1259.69** (719.99-2045.66)	15	1543.29** (863.73-2545.40)	11	1930.91** (963.87-3454.92)
Cardiovascular diseases	125	1.00 (0.84-1.20)	91	1.10 (0.89-1.36)	55	0.82 (0.62-1.07)	36	1.17 (0.82-1.62)
Digestive diseases	25	1.32 (0.85-1.95)	12	1.15 (0.60-2.01)	6	0.79 (0.29-1.71)	2	0.57 (0.07-2.07)
Accidents and violence	16	1.14 (0.65-1.85)	11	1.57 (0.79-2.81)	4	0.75 (0.20-1.91)	4	1.73 (0.47-4.43)

Supplementary table 5: Asbestos cement pooled Italian study. Mortality by gender and duration of employment for selected causes of death.

Obs.: observed; SMR: standardized mortality ratio; 95%CI: confidence intervals; * p < 0.05; ** p < 0.01.

MALES		≤ 1949		1950-9		1960-9		1970-9		1980-9		1990+
**Causes of death**	**n**	**SMR (95% CI)**	**n**	**SMR (95% CI)**	**n**	**SMR (95% CI)**	**n**	**SMR (95% CI)**	**n**	**SMR (95% CI)**	**n**	**SMR (95% CI)**
All causes	926	1.38** (1.30-1.48)	1679	1.24** (1.18-1.30)	2180	1.18** (1.13-1.23)	575	1.11* (1.02-1.21)	224	1.37** (1.20-1.57)	7	1.33 (0.53-2.73)
Malignant neoplasm	340	1.83** (1.64-2.03)	692	1.57** (1.46-1.69)	953	1.37** (1.28-1.46)	266	1.32** (1.16-1.48)	91	1.47** (1.19-1.81)	-	-
MN of stomach	22	1.08 (0.68-1.64)	28	0.73 (0.48-1.05)	58	0.97 (0.74-1.25)	19	1.16 (0.70-1.82)	9	1.93 (0.88-3.66)	-	-
MN of colon	22	1.76* (1.10-2.66)	29	1.09 (0.73-1.57)	45	0.99 (0.73-1.33)	14	1.03 (0.56-1.72)	7	1.68 (0.68-3.46)	-	-
MN of rectum	8	1.13 (0.49-2.23)	11	0.88 (0.44-1.58)	17	0.89 (0.52-1.42)	3	0.55 (0.11-1.59)	1	0.60 (0.02-3.37)	-	-
MN of peritoneum and retroperitoneum	23	26.88** (17.04-40.34)	46	25.28** (18.51-33.72)	29	9.04** (6.06-12.99)	4	4.06* (1.11-10.41)	-	-	-	-
MN of respiratory organs	155	2.69** (2.28-3.14)	381	2.45** (2.21-2.71)	484	1.96** (1.79-2.14)	121	1.71** (1.42-2.04)	43	2.06** (1.49-2.77)	-	-
MN of larynx	7	1.23 (0.49-2.53)	11	0.87 (0.43-1.55)	25	1.42 (0.92-2.10)	4	0.88 (0.24-2.26)	3	2.39 (0.49-6.99)	-	-
MN of the lung	112	2.26** (1.86-2.72)	273	1.99** (1.76-2.24)	324	1.47** (1.31-1.64)	84	1.33* (1.06-1.64)	27	1.44 (0.95-2.09)	-	-
MN of pleura	35	23.44** (16.33-32.61)	94	28.08** (22.69-34.36)	132	21.20** (17.74-25.14)	33	17.00** (11.70-23.88)	11	17.75** (8.86-31.77)	-	-
Respiratory diseases	189	3.34** (2.88-3.85)	222	2.14** (1.87-2.44)	228	2.00** (1.75-2.28)	31	1.16 (0.79-1.64)	9	1.37 (0.63-2.61)	-	-
Bronchitis, emphysema, asthma	17	0.60* (0.35-0.96)	54	1.11 (0.83-1.44)	67	1.65** (1.28-2.10)	9	1.15 (0.53-2.18)	3	1.85 (0.38-5.40)	-	-
Asbestosis	124	925.4** (769.6-1103.2)	120	520.2** (431.3-622.0)	94	365.1** (295.1-446.8)	9	180.4** (82.4-342.2)	1	72.8* (1.8-405.8)	-	-
Cardiovascular diseases	247	0.84** (0.74-0.95)	447	0.85** (0.78-0.94)	548	0.86** (0.79-0.94)	152	0.94 (0.80-1.11)	49	1.13 (0.84-1.50)	1	0.91 (0.02-5.09)
Digestive diseases	40	0.97 (0.69-1.32)	87	0.97 (0.78-1.20)	103	0.91 (0.74-1.11)	35	1.20 (0.84-1.68)	11	1.27 (0.64-2.28)	-	
Accidents and violence	32	1.25 (0.86-1.77)	32	0.60** (0.41-0.84)	100	1.03 (0.84-1.26)	35	0.92 (0.64-1.27)	19	0.98 (0.59-1.54)	3	2.82 (0.58-8.23)

FEMALES		≤ 1949		1950-9		1960-9		1970-9		1980-9		1990+
**Causes of death**	**n**	**SMR (95% CI)**	**n**	**SMR (95% CI)**	**n**	**SMR (95% CI)**	**n**	**SMR (95% CI)**	**N**	**SMR (95% CI)**	**n**	**SMR (95% CI)**
All causes	408	1.43** (1.29-1.57)	398	1.35** (1.22-1.49)	168	1.16 (0.99-1.35)	49	1.42* (1.05-1.88)	11	1.13 (0.56-2.02)	1	25.43 (0.64-141.70)
Malignant neoplasm	153	2.18** (1.85-2.55)	164	1.64** (1.40-1.91)	73	1.40** (1.09-1.76)	17	1.10 (0.64-1.76)	6	1.26 (0.46-2.75)	1	62.88* (1.57-350.39)
MN of stomach	6	1.05 (0.39-2.29)	9	1.43 (0.65-2.71)	4	1.12 (0.31-2.87)	2	2.26 (0.27-8.17)	1	3.85 (0.10-21.43)		-
MN of colon	10	1.58 (0.76-2.91)	12	1.50 (0.78-2.63)	4	0.94 (0.26-2.40)	-	-	-	-	-	-
MN of rectum	6	2.25 (0.83-4.90)	4	1.33 (0.36-3.39)	1	0.65 (0.02-3.64)	-	-	-	-	-	-
MN of peritoneum and retroperitoneum	20	31.16** (19.03-48.12)	9	11.04** (5.05-20.95)	1	2.29 (0.06-12.77)	1	8.37 (0.21-46.66)	-	-	-	-
MN of respiratory organs	39	6.07** (4.32-8.30)	55	5.09** (3.83-6.62)	29	4.77** (3.19-6.85)	5	2.51 (0.82-5.86)	2	3.21 (0.39-11.60)	-	-
MN of larynx	-	-	1	3.83 (0.10-21.32)	1	7.43 (0.19-41.39)	-	-	-	-	-	-
MN of the lung	9	1.65 (0.76-3.14)	21	2.20** (1.36-3.37)	7	1.28 (0.52-2.64)	-	-	1	1.75 (0.04-9.75)	-	-
MN of pleura	30	49.48** (33.38-70.63)	32	41.51** (28.40-58.60)	21	60.76** (37.61-92.87)	5	49.19** (15.97-114.79)	1	38.57 (0.96-214.92)	-	-
MN of the ovaries	8	2.43* (1.05-4.79)	7	1.32 (0.53-2.73)	3	1.04 (0.21-3.03)	-	-	-	-	1	1034.3** (25.9-5763.3)
Respiratory diseases	51	3.27** (2.44-4.30)	30	2.22** (1.50-3.17)	8	1.20 (0.52-2.37)	2	1.55 (0.19-5.61)	1	3.39 (0.09-18.92)	-	-
Bronchitis, emphysema, asthma	4	0.79 (0.22-2.03)	9	2.44* (1.12-4.63)	3	1.94 (0.40-5.68)	-	-	-	-	-	-
Asbestosis	33	1451.3** (999.0-2038.1)	14	772.4** (422.3-1295.9)	3	447.7** (92.4-1308.4)	1	541.5** (13.5-3017.3)	-	-	-	-
Cardiovascular diseases	126	0.93 (0.78-1.11)	110	1.01 (0.83-1.22)	53	1.05 (0.79-1.38)	16	1.79* (1.02-2.90)	2	1.05 (0.13-3.81)	-	-
Digestive diseases	10	0.72 (0.34-1.32)	19	1.11 (0.67-1.73)	14	1.93* (1.06-3.24)	2	1.18 (0.14-4.25)	-	-	-	-
Accidents and violence	10	1.01 (0.48-1.85)	8	0.78 (0.34-1.53)	10	1.73 (0.83-3.19)	6	3.23* (1.19-7.04)	1	1.27 (0.03-7.08)	-	-

Supplementary table 6. Asbestos cement pooled Italian study. Mortality by gender and period of first exposure for selected causes of death.
Obs.: observed; SMR: standardised mortality ratio; CI: confidence intervals; * $p < 0.05$; ** $p < 0.01$.
